# Around the Hospitals

**Published:** 1893-04-22

**Authors:** 


### Around the Hospitals.

Notice to the Managers of Institutions.—Special space is now reserved for the insertion of notices of hospital meetings and festivals. The Editor requests that all notices may reach The Hospital Office, 428, Strand, at least one week before the date of each meeting.

**Poplar Hospital for Accidents.**—It was a pleasant announcement that was made at the recent annual meeting of this hospital. During the year no less than £4,158 has been contributed towards the much-needed ward for women. This addition will make the work of the hospital far more complete. In such a neighbourhood as Poplar accidents are not confined to the one sex alone, and the want of help for the female population has been much felt. Contributions towards the general work of the hospital have been generously made, and large legacies have been received. Institutions situated as is the Poplar Hospital are only too easily overlooked, and the greatest energy on the part of the management is necessary to obtain just recognition for much useful work. The institution is fortunate in possessing good friends such as Hon. Sydney Holland and other indefatigable workers on its behalf.

**The Royal Infirmary, Newcastle.** An experiment is being emade at the present time of a house to house collection in aid of this institution. It may prove useful to the management of other hospitals to watch the result of this effort. At the present time nearly £300 as been contributed, and increased annual subscriptions are reported. The report for 1892 shows a lower death-rate, a diminution in the cost per patient (£52 13s. 7½d.) in the whole expenditure, in the debt, in the expenditure on provisions, drugs, surgical instruments, and a decrease in the amount subscribed by working classes. Increase is shown in the number of subscribers; in the cost of furniture and repairs; and of ale, wine, and spirits. Although legacies have not been received for general purposes to the present time, intimation has been received of several large legacies in prospect. The Committee express the general regret felt at the loss of the chairman, the Rev. John Collingwood Bruce. They express their appreciation of the services of the nursing staff under the Lady Superintendent, Miss Agnes Ross. We congratulate Mr. William Oliver, the secretary, on the creditable manner in which he has drawn up the report, one special feature of which is a report by the Chaplain of the infirmary. The long list of donors of old linen, flowers, and papers show that a great interest is taken locally in the institution.

**The Worcester General Infirmary** is amongst the large number of hospitals that have to report a decrease in the amount of annual subscriptions. On the other hand considerable sums derived from legacies have fallen to the share of this institution during the past year. The largest of these is a bequest from Mr. Charles Ganderton, of Pershore, amounting to £7,000, and the residue of his estate after the payment of other legacies. Mr. Ganderton presented £5,000 to the hospital anonymously on a previous occasion. In memory of these munificent gifts a ward is to be named after this gentleman. At the recent annual meeting it was decided to admit working men, members of the Hospital Saturday Fund, to become governors of the infirmary. The rules were also altered to provide for the appointment of the house surgeon for three, and the assistant house surgeon for two years.

